# DYNAMIC EFFECTS OF DIETARY PRUNE ON THE GUT-BONE AXIS IN HEALTHY FEMALE MICE

**DOI:** 10.51894/001c.122798

**Published:** 2024-08-30

**Authors:** Nicholas Chargo, Kavisha Patel, Shashank Ravishankar, Leon Regnery, Douglas V. Guzior, Joseph D. Gardinier, Robert A. Quinn, Narayanan Parameswaran, Laura R. McCabe

**Affiliations:** 1 College of Osteopathic Medicine Michigan State University

## O3

### INTRODUCTION

The incidence of low bone mass and osteoporosis worldwide is increasing. Novel treatment options to combat further bone loss and improve peak bone mass prior to age or disease related decline are highly desirable. Dietary prune (DP) supplementation, a gut-targeted therapy, is an effective preventative for primary osteoporosis in rodents and humans. However, DP effects on the gut-bone axis are not well described in healthy female animals.

### OBJECTIVES/HYPOTHESIS

The goal of this study was to understand the effects of continual and intermittent feeding of DP on the gut-bone axis in healthy female mice. We hypothesized that DP would lead to highly dynamic, time-dependent improvements in gut and bone health that would be maintained with intermittent feeding.

### METHODS

Sixteen-week-old female C57BL/6J mice (n =10/group) were fed AIN-93M control (C) or 25% prune supplemented diet (25%P) continually for periods of 5-days to 16-weeks. There were also groups fed 25%P for 4-weeks followed by 1-, 2-, or 3-weeks C to determine the effects following discontinuation. Finally, two groups were fed 25%P for 4-weeks, switched to C for 1-week, then readministered 25%P for 1- or 2-weeks. Femur trabecular microarchitecture, tibial biomechanics, gut microbiota composition, and intestinal barrier function were assessed.

## RESULTS

Continual feeding of 25%P increased trabecular bone density as early as 5-days (30%, p<0.0001) with time-dependent increases through 16-weeks (220%, p<0.0001). This was accompanied by functional improvements in tibial biomechanical properties at 16-weeks. In the gut, 25%P altered gut microbiota composition at all timepoints (p=0.001) and intestinal barrier function was improved as early as 5-days (p<0.0001). Intermittent feeding of 25%P revealed that bone and intestinal barrier benefits were lost after 2-3 weeks of discontinuation, however, could be restored with 1-week 25%P. Gut microbiota composition was partially maintained after discontinuation and intermittent feeding of 25%P.

### DISCUSSION/CONCLUSION

These results demonstrate the dynamic effect of DP on the gut-bone axis in healthy female mice. Continual feeding results suggest that continual DP supplementation may help attain peak bone mass and improve overall gut health. The intermittent feeding results suggest that DP can be consumed intermittently and still maintain their beneficial effect on the gut-bone axis.

